# Metavirome Analysis of *Culex tritaeniorhynchus* Reveals Novel Japanese Encephalitis Virus and Chikungunya Virus

**DOI:** 10.3389/fcimb.2022.938576

**Published:** 2022-06-30

**Authors:** Duo Zhang, Chengcheng Peng, Chenghui Li, Yiquan Li, He Zhang, Nan Li, Pengpeng Xiao

**Affiliations:** ^1^ Wenzhou Key Laboratory for Virology and Immunology, Institute of Virology, Wenzhou University, Wenzhou, China; ^2^ College of Agriculture, Yanbian University, Yanji, China; ^3^ Academician Workstation of Jilin Province, Changchun University of Chinese Medicine, Changchun, China; ^4^ Changchun Veterinary Research Institute, Chinese Academy of Agricultural Sciences, Changchun, China

**Keywords:** metavirome analysis, *Culex tritaeniorhynchus*, viral isolation, virus identification, phylogenetic analysis

## Abstract

To explore the *Culex tritaeniorhynchuses–*specific virome, 6400 C*. tritaeniorhynchuses* were collected in Honghe autonomous prefecture, China. Abundant virus sequences were obtained from 28 viral families using metavirome sequencing. Herein, several viruses in *C. tritaeniorhynchuses* virome were verified using the PCR technique, which covers Japanese encephalitis virus (JEV), Getah virus, and even Chikungunya virus (CHIKV). Seven JEV gene sequences were amplified successfully, of which JEV-China/CT2016E-1 shared the highest homology with the known JEV sequence isolated in Korea, 1946, with at least 96.1% nucleotide (nt) identity, which belonged to genotype III. Nine CHIKV gene sequences were amplified, which shared the highest with at least 93.0% nt identity with CHIKV from Thailand isolated in 2007, which was assigned to genotype Asian. Remarkably, CHIKV was isolated from *C. tritaeniorhynchus* in China for the first time. It was initially confirmed that the isolated virus CHIKV-China/CT2016-1 may increase infectivity after passaging in Vero cells from BHK-21 cells. Collectively, our study reveals the diversity, properties, and potential virus susceptibility dynamics of the *C. tritaeniorhynchus* virome and sheds new perspectives on the viral ecology in other important biological vectors.

## Introduction

Mosquitoes are the biological vectors of many important zoonotic viruses, which can not only infect animal, plants, and fungi but also pose a serious threat to human life and health (Lumley et al., 2018; Sarah et al., 2021). China’s Yunnan province is located at the junction of China, Myanmar, Laos, and Vietnam and has a tropical rainforest climate, which is rich in mosquito-borne viruses (Chao et al., 2017; Rui-Chen et al., 2020). Notably, *Culex tritaeniorhynchus* is the dominant mosquito species in the region (Yuan et al., 2021). Therefore, the surveillance of *C. tritaeniorhynchus–*specific viruses is of great value. Traditional methods applied for virus detection, such as RT-PCR (Reverse Transcription-Polymerase Chain Reaction) and WB (Western blotting), can accurately identify several viruses (Micah et al., 2021; Ptasinska et al., 2021). However, considering the detection of a large number of low-abundance unknown viruses, metavirome sequencing has more advantages (Jenai et al., 2019). Furthermore, viral metagenomics offers a great opportunity for the bulk analysis of viral genomes retrieved directly from environmental samples (Muddassar et al., 2020). The development of metavirome sequencing technologies have resulted in an explosion in the identification of viruses, with over 2,000 novel viruses recorded in recent years (Mang et al., 2016; Haoming et al., 2020). Accordingly, the application of metavirome sequencing to *C. tritaeniorhynchus* can effectively avoid the missed detection of highly pathogenic viruses and potential unknown viruses.

The purpose in our study was to explore the *C. tritaeniorhynchus–*specific viruses in Yunnan province and to provide valuable reference for the discovery, isolation, and identification of new viruses. We found that there were abundant virus species in *C. tritaeniorhynchus* in Yunnan province using metavirome sequencing. Apart from new variants of JEV and Getah virus (GETV), Chikungunya virus (CHIKV) was isolated in *C. tritaeniorhynchus* from China for the first time. Our preliminary study on the metavirome of *C. tritaeniorhynchus* provides valuable information for the discovery, diversity, and monitoring of the virome in other mosquito species.

## Material and Methods

### Mosquito Collection

**Figure 1:**
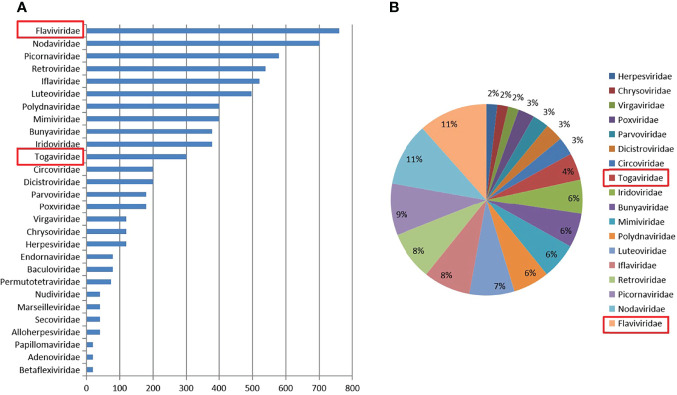
The sample collection site in Yunnan province, China. The sample collection site was labeled with a black mosquito pattern.

Female mosquitoes were collected daily from barns in Honghe autonomous prefecture (N 23° 37’, E 102° 42’), (**Figure 1**) China, from May to July 2016. Mosquitoes were collected through a mosquito trap lamp played overnight from 17:00 to 08:00. After retrieval, the collected mosquitoes were placed on an ice surface and loaded mosquitoes were morphologically identified as *C. tritaeniorhynchus* into 2-ml U-bottom microcentrifuge tubes with a maximum of 100 mosquitoes per tube and then stored at −80°C.

### RNA Extraction and Metaviral Sequencing

Reagents together with methods used in RNA extraction and metaviral sequencing have been expounded in our previous study (Feng et al., 2022). Moreover, the DNA barcodes employed in metaviral sequencing are displayed in **Table 1**. To identify viral sequences, blastn (https://blast.ncbi.nlm.nih.gov/Blast.cgi) was employed for contig comparison to the GenBank, in which the E value ≤10e^−5^ was incorporated into the adoption. To further validate the metaviral sequencing results, RT-PCR was applied for confirming viral sequences using specific primers (**Table 2**).

**Table 1:** Barcode DNA employed in metagenomic analysis (Pengpeng et al., 2018).

Primer Type	Primer Number	Primers (5’-3’)
Anchored Random Primers	RT1	GCCGGAGCTCTGCAGATATCNNNNNN
	RT 2	GTATCGCTGGACACTGGACCNNNNNN
	RT 3	ATCGTCGTCGTAGGCTGCTCNNNNNN
Barcode Primers	Primer1	GCCGGAGCTCTGCAGATATC
	Primer2	GTATCGCTGGACACTGGACC
	Primer3	ATCGTCGTCGTAGGCTGCTC

**Table 2:** Primer pairs used in PCR identification.

Primer Name	Primers (5’-3’)	Product (bp)
JEV-China/CT2016E-1/2/3-F	TTTAATTGTCTGGGAATGGGCA	1,500
JEV-China/CT2016E-1/2/3-R	AGCATGCACATTGGTCGCTAAGA	
JEV-China/CT2016NS1-1/2/3/4-F	GACACTGGATGTGCCATTGACA	1,056
JEV-China/CT2016NS1-1/2/3/4-R	AGCATCAACCTGTGATCTAACGA	
CHIKV-China/CT2016E1-1/2/3/4-F	TACGATCAGGTAACTGTGAACC	1,317
CHIKV-China/CT2016E1-1/2/3/4-R	GTGCCTGCTAAACGACACGCATAG	
CHIKV-China/CT2016C-1/2/3/4/5-F	ATGGAGTTCATCCCAACCCAAA	783
CHIKV-China/CT2016C-1/2/3/4/5-R	CCACTCTTCGGCCCCCTCG	
GETV-China/CT2016E1-1/2/3-F	TACGAACACACCGCAACGATC	1,314
GETV-China/CT2016E1-1/2/3-R	GCGGCGCATAGTCACACACG	
CHIKV-F*	CTATGGAGCCAACGCTATCGCT	326
CHIKV-R*	AGATACAGTAACATTATTTCCT	

*The primers used in CHIKV identification after viral isolation.

### Phylogenetic Analysis

The complete viral genes amplified were aligned with representative viral genes through Clustal W 2.0. The maximum-likelihood algorithm was utilized for the tree-building model using MEGA 7.0.

### Virus Isolation and Identification

BHK-21 and Vero cells were applied for virus isolation using DMEM (Dulbecco's modified Eagle's medium)(HyClone, Logan, UT, USA) with 8% fetal bovine serum added. In a nutshell, after centrifugation, 20 μl of the supernatant of the mosquito-grinding solution was added to the monolayer of BHK-21 cells, on which 80 μl of DMEM was contained and incubated at 37°C for 6 days until the cells were observed to have a cytopathic effect (CPE) induced by the virus. Blind passage was performed for 3 times when no CPE appeared at the first time. CHIKV was searched in supernatants after virus isolation using CHIKV-specific primers (Table 2) targeting 326 nt of the E1 gene. Furthermore, the expression of E1 protein was tested through Western blotting with the anti-E1 monoclonal antibody (Abcam, Cambridge, UK) and an HRP-conjugated rabbit anti-mouse antibody (Trans, Beijing, China). Additionally, negative-stained electron microscopy was employed for the observation of CHIKV particles, which were prepared using supernatant of infected BHK-21 cells mingled 1:1 with 6.1% paraformaldehyde, mounted on copper grids, with the treatment of 3% phosphotungstic acid.

### Viral Passage and Variation Analysis

To mimic natural infection at the cellular level, we passaged the CHIKV in the susceptible cells including Vero cells and BHK-21 cells. Firstly, CHIKV was passaged in BHK cells for 10 times to get the 10th generation, and then, the virus was harvested from the supernatants of BHK cells infected in the 10th-generation CHIKV, followed by inoculation into Vero cells for passaging another 10 times, getting a total of 20 generations of CHIKV. The TCID_50_ of the 5th and 10th passages from BHK and the 15th and 20th from Vero cells was tested according to the Reed−Muench method (Krah, 1991). Subsequently, the E1 gene of the above four passages was sequenced, with alignment using MEGA 7.0.

### Statistical Analysis

Data analysis was performed through SPSS (Statistical Product Service Solutions) 19.0, in which a comparison between groups was performed using the T-test. Each experiment was validated iteratively by setting up three replicates and repeating it three times. Differences were considered significant when the P-value was less than 0.05.

### Data Availability

The data produced in metagenomic sequencing have been deposited in the GenBank Sequence Reads Archive with the accession number of SRR15291873. The amplified viral sequences have been deposited in the GenBank, in which the accession numbers were covered: JEV-China/CT2016E-1/2/3(OM799545–OM799547), JEV-China/CT2016NS1-1/2/3/4 (OM799554–OM799557), GETV-China/CT2016E1-1/2/3 (OM799565–OM799567), CHIKV-China/CT2016E1-1/2/3/4 (OM799570–OM799573), and CHIKV-China/CT2016C-1/2/3/4/5 (OM799581–OM799583, OM898920, OM799584), respectively.

## Results

### Mosquito Virome

**Figure 2:**
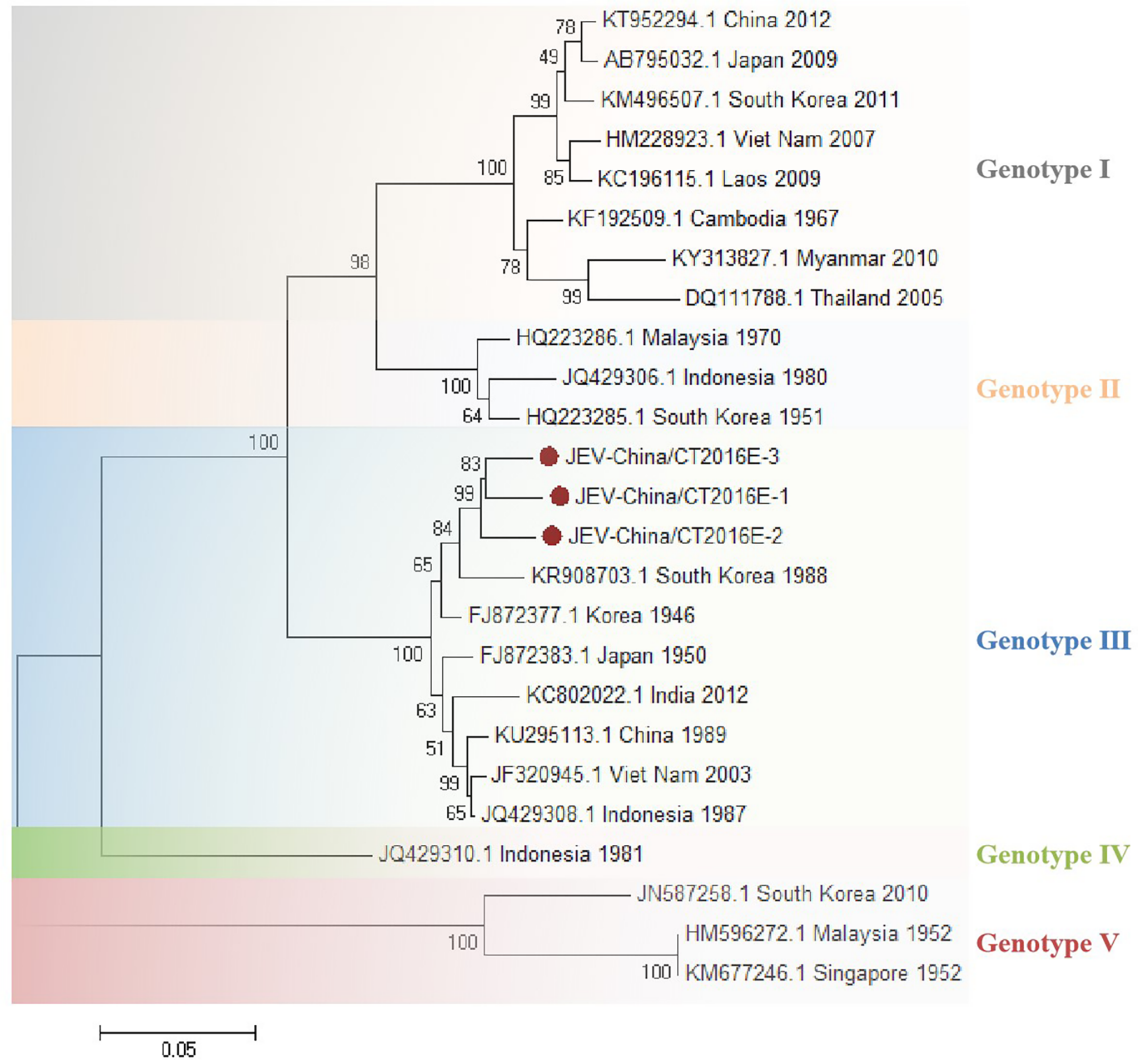
Abundance and proportion of viral families in *Culex tritaeniorhynchus*. The classification of virus sequences according to the viral family, with relative abundance **(A)**. The proportion of viral families in *C. tritaeniorhynchus* **(B)**.

Approximately 6,400 mosquitoes classified as *C. tritaeniorhynchus* were collected for metaviral sequencing. Sequences were sorted as viruses with 7.02 × 10^6^ reads, including known and unclassified viruses. Many mosquito viruses together with unknown or unclassified viruses were discovered, with potential infectious risks to human and animal. The abundance of viral reads varied largely by the virus taxonomy. A total of 28 viral families were detected, (**Figure 2A**) among which are *Flaviviridae* and *Togaviridae*, consisted of known vital human pathogens verified in *C. tritaeniorhynchus.* (**Figure 2B**) Metaviral sequencing assay results showed that *C. tritaeniorhynchus* can carry a variety of potential zoonotic pathogens. A total of 23,347 contigs were yielded from the *de novo* assembly. Some interesting assembled contigs showed similarities to the viral sequences of *Flaviviridae* and *Togaviridae*, among which 127 JEV-like contigs, 59 GETV-like contigs, and 92 CHIKV-like contigs demonstrated reads coverage of 34× (261–1035 nt), 25× (239–916 nt), and 37× (341–2,416 nt), respectively. Moreover, the JEV-like contigs, GETV-like contigs, and CHIKV-like contigs possessed 83.2%–95.7%, 82.6%–96.4%, and 81.9%–97.3% nt homology with the known JEV, GETV, and CHIKV sequences separately. Subsequently, virus-specific primers (**Table 2**) were designed and synthesized for the amplification of the detected virus-like sequences, by which the results from metaviral sequencing were verified.

### The Verification of Japanese Encephalitis Virus–Like Sequences

**Figure 3:**
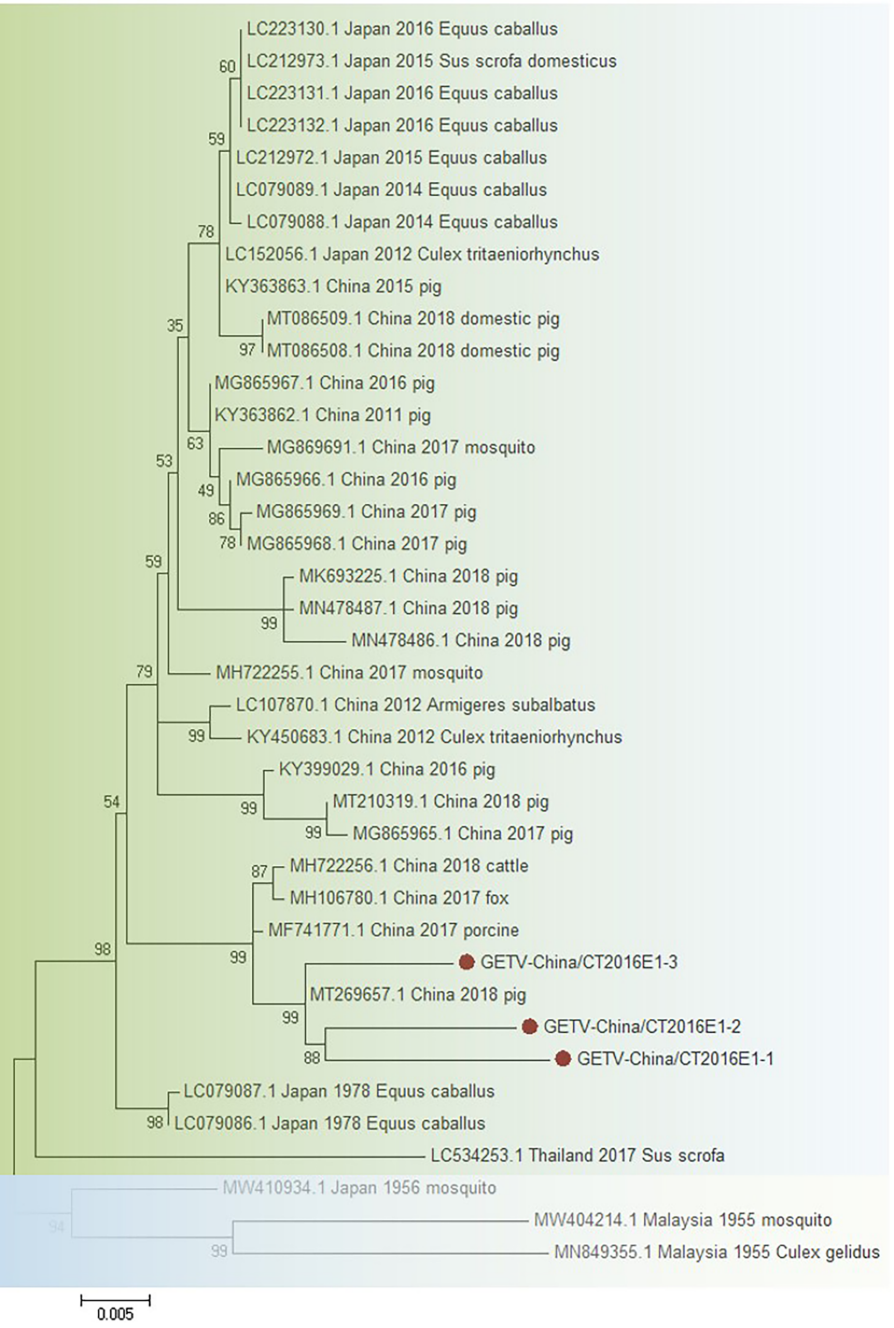
The phylogenetic trees based on JEV-E. E gene of JEV used to build phylogenetic trees. Application of the maximum likelihood method was used in MEGA 7.0, in which the bootstrap value was set to 1,000. Solid red circles were used to mark the viral genes that were amplified in this study.

The amplified JEV-like fragments were cloned into the pMD19-T vector (Takara, Tokyo, Japan), followed by sequencing. Three 1,500-bp fragments (JEV-China/CT2016E-1/2/3) and four 1,056-bp fragments (JEV-China/CT2016NS1-1/2/3/4) were obtained. After alignment, JEV-China/CT2016E-1/2/3 shared ~96.5%–96.7% nt identity and ~90.6%–92.6% amino acid (aa) identity with one another. Additionally, JEV-China/CT2016NS1-1/2/3/4 possessed ~95.3%–96.6% nt identity and ~88.1%–91.5% aa identity between each other. Viral sequence analysis using blastn showed that the amplified JEV-like fragments shared the highest (nt identity at least 96.1% and aa identity at least 94.2%) identity with JEV from Korea isolated in 1946, which was assigned to genotype III JEV (**Figure 3**).

### The Verification of Getah Virus–Like Sequences

**Figure 4:**
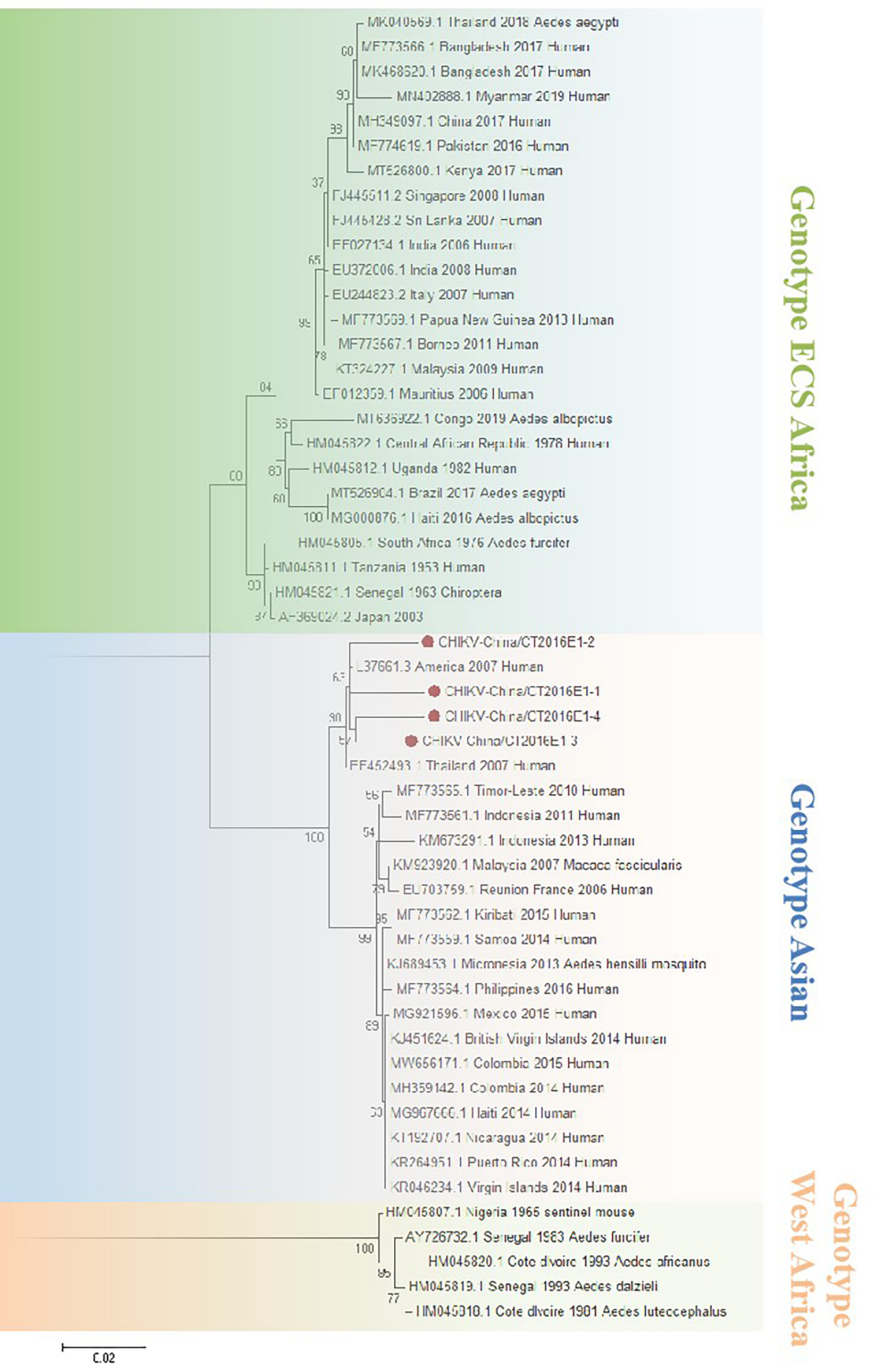
The phylogenetic trees based on GETV-E1. E1 gene of GETV used to build phylogenetic trees. Application of the maximum likelihood method was used in MEGA 7.0, in which the bootstrap value was set to 1,000. Solid red circles were used to mark the viral genes that were amplified in this study.

The amplified GETV-like fragments were cloned into the pMD19-T vector (Takara, Tokyo, Japan), followed by sequenced. Three 1,314-bp fragments (GETV-China/CT2016E1-1/2/3) were acquired. After alignment, GETV-China/CT2016E1-1/2/3 shared ~97.0%–97.4% nt identity and ~92.0%–93.8% aa identity with one another. Viral sequence analysis using blastn showed that the amplified GETV-like fragments shared the highest (nt identity at least 98.2% and aa identity at least 94.3%) identity with the E1 gene of GETV from Japan isolated in *Equus caballus*, 1978 (**Figure 4**).

### The Verification of Chikungunya Virus–Like Sequences

**Figure 5:**
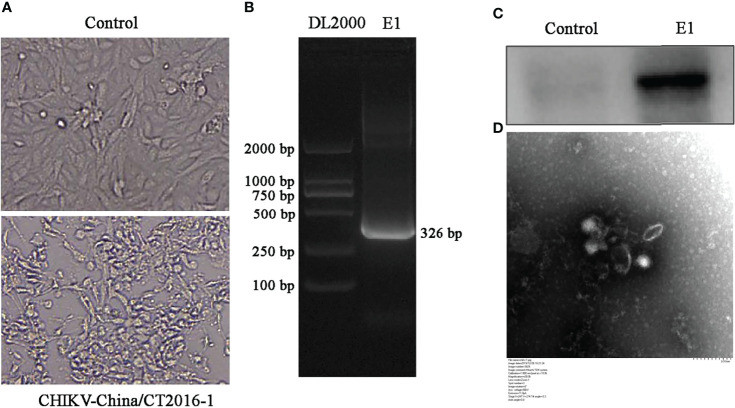
The phylogenetic trees based on CHIKV-E1. E1 gene of CHIKV used to build phylogenetic trees. Application of the maximum likelihood method was used in MEGA 7.0, in which the bootstrap value was set to 1,000. Solid red circles were used to mark the viral genes that were amplified in this study.

The amplified CHIKV-like fragments were cloned into the pMD19-T vector (Takara, Tokyo, Japan), followed by sequencing. Four 1,317-bp fragments (CHIKV-China/CT2016E1-1/2/3/4) and five 783-bp fragments (CHIKV-China/CT2016C-1/2/3/4/5) were obtained. After alignment, CHIKV-China/CT2016E1-1/2/3/4 shared ~96.5%–97.3% nt identity and ~90.9%–93.8% aa identity with one another. Additionally, CHIKV-China/CT2016C-1/2/3/4/5 possessed ~92.0%–97.2% nt identity and ~91.2%–94.6% aa identity between each other. Viral sequences analysis using blastn showed that the amplified CHIKV-like fragments shared the highest (nt identity at least 93.0% and aa identity at least 94.3%) identity with CHIKV from Thailand isolated in 2007, which was assigned to genotype Asian (**Figure 5**).

### Viral Isolation Identification of Chikungunya Virus

**Figure 6:**
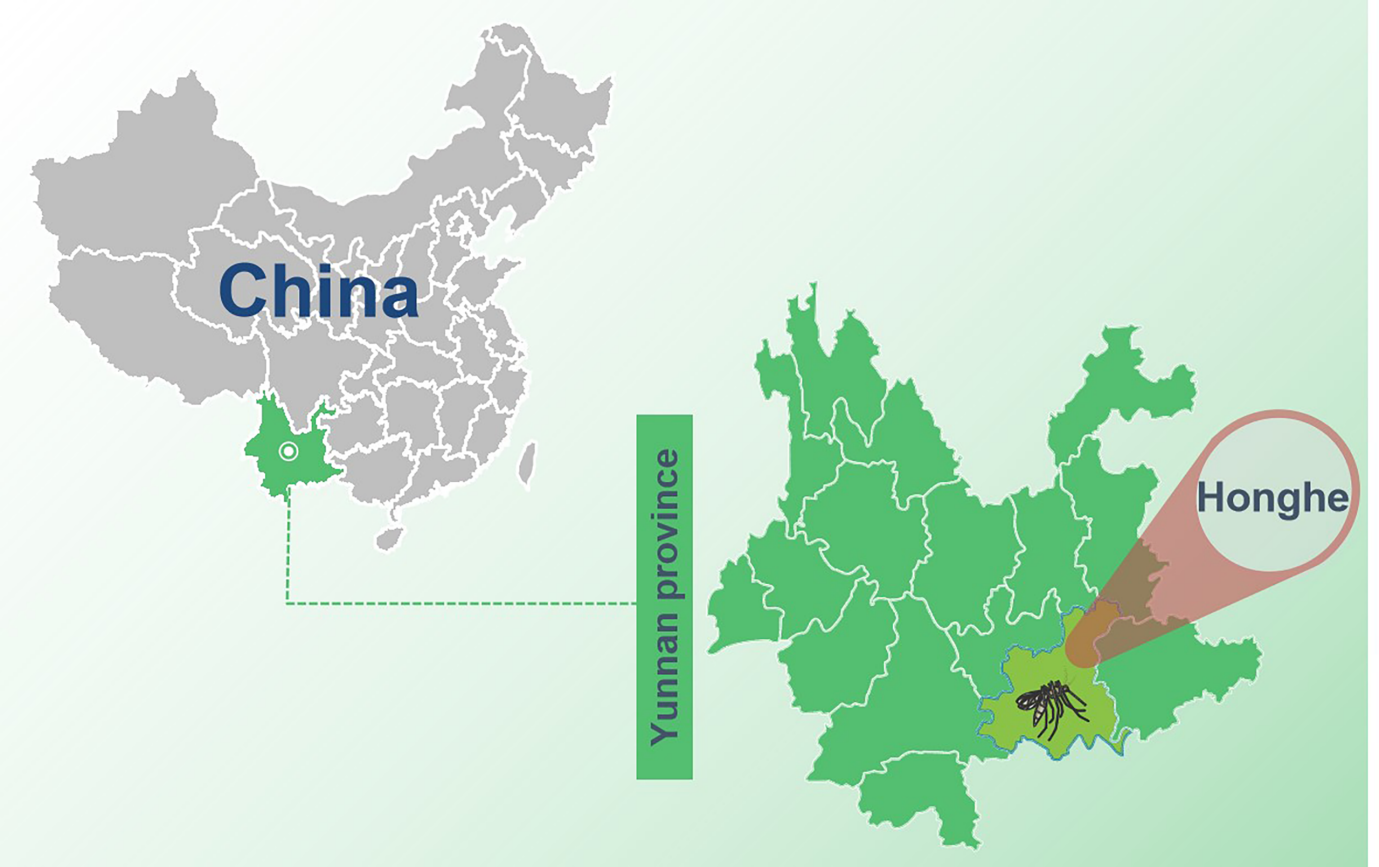
Identification of *CHIKV-China/CT2016-1* isolated in the Honghe autonomous prefecture of Yunnan province. CPE observation of *CHIKV-China/CT2016-1* after BHK-21 cell infection **(A)**. Identification of *CHIKV-China/CT2016-1* by PCR **(B)**. Identification of *CHIKV-China/CT2016-1* by Western blot **(C)**. Negative-stain electron microscopy of *CHIKV-China/CT2016-1* particles **(D)**.

All validated viruses were attempted to be isolated, and only CHIKV was successfully isolated. The newly isolated CHIKV was verified by CPE (**Figure 6A**), PCR at the gene level (**Figure 6B**), and WB at the protein expression level in infected cells (**Figure 6C**), and the results all demonstrated positive. To more visually verify the newly classified CHIKV, we observed virus particles by negative-stain electron microscopy, displaying rounded with belike 70-nm diameter and tiny protrusions on the viral surface (**Figure 6D**).

### The Variability of Chikungunya Virus After Consecutive Passages

The infectious titer value of CHIKV-China/CT2016-1 at the 5th generation in BHK cells was assessed as 6.35 × 10^4^ TCID_50_/0.1 ml, and that at the 10th generation was tested as 5.73 × 10^4^ TCID_50_/0.1 ml. The 10th generation showed a decrease in infectivity compared to the 5th generation, but did not show significant differences. Interestingly, after examed in Vero cells, the infectious titre value presented 2.26 × 10^5^ TCID_50_/0.1 ml at the 15th generation and 3.03 × 10^5^ TCID_50_/0.1 ml at the 20th generation, respectively. Both of the 15th and 20th generations showed a significant increase in infectivity compared to the 5th and 10th generations; thus, it can be speculated that cross-species transmission may have contributed to the increased infectivity of the virus. Further sequence analysis showed that both the E1 genes of the 15th and 20th generations had mutation in a key aa site (A226V) compared to the 5th and 10th generations.

## Discussion

Mosquitoes are the biological vectors for the transmission of numerous important viruses, which are not only harmful to plants and animals but also a serious threat to human life (Laura et al., 2019; Priscilla et al., 2020; Chasity et al., 2022). In our previous study, we found that *C. tritaeniorhynchus* was the dominant mosquito species in Yunnan province, China (Xiao et al., 2018). Multiple studies found that *C. tritaeniorhynchus* carried a rich variety of viruses, covering Zika virus (Jing et al., 2021), Duck Tembusu virus (Jitra et al., 2021), and JEV (Astri et al., 2021). In this study, a preliminary exploration of the viral spectrum in the *C. tritaeniorhynchus* from Yunnan province was carried out using metavirome techniques, and rich viral sequences were detected, belonging to 24 viral families. Our study offers support for a focus on the breadth of the viral spectrum of the major mosquito species circulating in this region. In addition, it highlights the importance of conducting vector-specific surveillance to detect mosquito-borne viruses of human-wide health importance.

Validation based on metaviral sequencing results, JEV and GETV were successfully amplified from *C. tritaeniorhynchus*, indicating that JEV and GETV were potentially propagated and circulated in this area. Furthermore, CHIKV was isolated. To the best of our knowledge, this is the first report of CHIKV isolation from *C. tritaeniorhynchus* in China. This finding supported the evidence once again for CHIKV transmission in *C. tritaeniorhynchus* (Hasan et al., 2020), suggesting that CHIKV was at risk of potentially expanding the susceptible mosquito species and thus the geographical range of transmission. Moreover, CHIKV and JEV were detected simultaneously in the same mosquito species and geographic location, demonstrating that CHIKV and JEV existed cocirculation, the role of which in the expansion of the range of CHIKV-susceptible mosquito species needed to be further investigated. *Aedes agypti* and *Ae. albopictus* have been implicated as the primary natural transmission vectors of CHIKV. The productive arboviral infection in competent mosquito vector involves the infection of the midgut and spread within the midgut epithelium followed by dissemination to secondary tissues where viral amplification takes place and infects salivary glands to release the virus into salivary ducts necessary for infection to the vertebrate host (Hardy et al., 1984). The detection of CHIKV in *Cx. tritaeniorhynchus* and isolation of virus in susceptible cell line signify infection with CHIKV, but to establish the vectorial capabilities, further research on vector competence of the mosquito is necessary. Moreover, to support appropriately the isolation of mosquito-transmitted arboviruses, other cell lines of the mosquito origin (e.g., C6/36, C7-10, CCL-125, and Age-2) could be used in future experiments. In addition, some viruses were not amplified, probably due to insufficient sample size or low viral load.

The variability analysis of CHIKV showed that the E1 genes of the 15th and 20th generations had mutation in a key aa site (A226V) compared to the 5th and 10th generations. As reported, E1-A226V mutation will significantly enhance the adaptability of CHIKV in *Ae. albopictus* (Agbodzi et al., 2021). In our study, both of the 15th and 20th generations showed a significant increase in infectivity compared to the 5th and 10th generations, suggesting that CHIKV may have the potential to propagate across the species barrier with infectivity enhancement. This meant that the continued spread of CHIKV will pose a greater potential threat to human health.

The findings in this study demonstrated the potential viral reservoir in the *C. tritaeniorhynchus* in this region. However, the specific viral profile of *C. tritaeniorhynchus* is incomplete. Unknown viruses of specific mosquito species need to be revealed through further research. In conclusion, our study provides valuable insights into the specific mosquito-borne virus spectrum and the excavation of its new susceptible viruses. This will provide an important reference value for the molecular epidemiological studies of arboviruses.

## Data Availability Statement

The datasets presented in this study can be found in online repositories. The names of the repository/repositories and accession number(s) can be found in the article/supplementary material.

## Author Contributions

PX and NL conceived and designed the experiments. DZ, CP, CL, and HZ performed the experiments. PX and YL analyzed the data. CL contributed reagents/materials/analysis tools. PX and NL wrote the paper. All authors read and approved the final version of the manuscript.

## Funding

This work was supported by the National Natural Science Foundation of China [grant number 32002312], the Natural Science Foundation of Zhejiang Province [grant number LQ21H160001], Science and Technology Project of Wenzhou, Zhejiang, China [grant number Y20210080 and Y2020103].

## Conflict of Interest

The authors declare that the research was conducted in the absence of any commercial or financial relationships that could be construed as a potential conflict of interest.

## Publisher’s Note

All claims expressed in this article are solely those of the authors and do not necessarily represent those of their affiliated organizations, or those of the publisher, the editors and the reviewers. Any product that may be evaluated in this article, or claim that may be made by its manufacturer, is not guaranteed or endorsed by the publisher.

## References

[B1] Agbodzi B. Yousseu F. Simo F. Kumordjie S. Yeboah C. Mosore M. T.. (2021). Chikungunya Viruses Containing the A226V Mutation Detected Retrospectively in Cameroon Form a New Geographical Subclade. Int. J. Infect. Dis. 113, 65–73. doi: 10.1016/j.ijid.2021.09.058 34592442

[B2] Astri N. F. Daisuke K. Michael A.-B. Yukiko H. Yoshio T. Kentaro I.. (2021). Evaluating the Competence of the Primary Vector, Culex Tritaeniorhynchus, and the Invasive Mosquito Species, Aedes Japonicus Japonicus, in Transmitting Three Japanese Encephalitis Virus Genotypes. PLoS. Negl. Trop. Dis. 14 (12), e0008986. doi: 10.1371/journal.pntd.0008986 PMC7793266 33370301

[B3] Chao W. Xiaofang G. Jun Z. Quan L. Hongbin L. Edward B. M.. (2017). Behaviors Related to Mosquito-Borne Diseases Among Different Ethnic Minority Groups Along the China-Laos Border Areas. Int. J. Environ. Res. Public. Health 14 (10), 1227. doi: 10.3390/ijerph14101227 PMC5664728 29036937

[B4] Chasity E. T. Gabriela R. Irma S.-V. Laura A. S. C. Oshani C. R. Shirley L.. (2022). Coupled Small Molecules Target RNA Interference and JAK/STAT Signaling to Reduce Zika Virus Infection in Aedes Aegypti. PLoS. Pathog. 18 (4), e1010411. doi: 10.1371/journal.ppat.1010411 35377915 PMC9017935

[B5] Feng G. Zhang J. Zhang Y. Li C. Zhang D. Li Y.. (2022). Metagenomic Analysis of Togaviridae in Mosquito Viromes Isolated From Yunnan Province in China Reveals Genes From Chikungunya and Ross River Viruses. Front. Cell. Infect. Microbiol. 12. doi: 10.3389/fcimb.2022.849662 PMC8878809 35223559

[B6] Haoming W. Rui P. Tong C. Liang X. Haiyan Z. Tao L.. (2020). Abundant and Diverse RNA Viruses in Insects Revealed by RNA-Seq Analysis: Ecological and Evolutionary Implications. mSystems 5 (4), e00039–e00020. doi: 10.1128/mSystems.00039-20 32636338 PMC7343303

[B7] Hardy J. L. Rosen L. Reeves W. C. Scrivani R. P. Presser S. B. (1984). Experimental Transovarial Transmission of St. Louis Encephalitis Virus by Culex and Aedes Mosquitoes. Am. J. Trop. Med. Hyg. 33 (1), 166–175. doi: 10.4269/ajtmh.1984.33.166 6696174

[B8] Hasan B. Laurence M. Sara M. Marie V. Géraldine P. Sedigheh Z.. (2020). Detection of Arboviruses in Mosquitoes: Evidence of Circulation of Chikungunya Virus in Iran. PLoS. Negl. Trop. Dis. 14 (6), e0008135. doi: 10.1371/journal.pntd.0008135 32603322 PMC7357783

[B9] Jenai Q. Charles L. Alison K. Joshua B. Noam T. Amy L.. (2019). FLASH: A Next-Generation CRISPR Diagnostic for Multiplexed Detection of Antimicrobial Resistance Sequences. Nucleic. Acids Res. 47 (14), e83. doi: 10.1093/nar/gkz418 31114866 PMC6698650

[B10] Jing W. Hongbin X. Song S. Rui C. Na F. Shihong F.. (2021). Emergence of Zika Virus in Culex Tritaeniorhynchus and Anopheles Sinensis Mosquitoes in China. Virol. Sin. 36 (1), 33–42. doi: 10.1007/s12250-020-00239-w 32617898 PMC7973324

[B11] Jitra S. Nichapat Y. Aunyaratana T. Sonthaya T. (2021). Vector Competence of Culex Tritaeniorhynchus and Culex Quinquefasciatus (Diptera: Culicidae) for Duck Tembusu Virus Transmission. Acta. Trop. 214, 105785. doi: 10.1016/j.actatropica.2020.105785 33309596

[B12] Krah D. L. (1991). A Simplified Multiwell Plate Assay for the Measurement of Hepatitis A Virus Infectivity. Biologicals 19 (3), 223–237. doi: 10.1016/1045-1056(91)90039-m 1659431

[B13] Laura R. H. A. Chasity E. T. Grace F. C. Sophie M. Brandi K. T. Clement Y. C.. (2019). Insulin Potentiates JAK/STAT Signaling to Broadly Inhibit Flavivirus Replication in Insect Vectors. Cell. Rep. 29 (7), 1946–1960.e5. doi: 10.1016/j.celrep.2019.10.029 31722209 PMC6871768

[B14] Lumley S. Hernández-Triana L. M. Horton D. L. Fernández de M. M. Medlock J. M. Hewson R.. (2018). Competence of Mosquitoes Native to the United Kingdom to Support Replication and Transmission of Rift Valley Fever Virus. Parasitol. Vectors 11 (1), 308. doi: 10.1186/s13071-018-2884-7 PMC5960175 29776384

[B15] Mang S. Xian-Dan L. Jun-Hua T. Liang-Jun C. Xiao C. Ci-Xiu L.. (2016). Redefining the Invertebrate RNA Virosphere. Nature 540 (7634), 539–543. doi: 10.1038/nature20167 27880757

[B16] Micah T. M. Florica J. C. Bradly P. N. Marshall N. Thomas W. B. Ricardo H.. (2021). A Blood-Based Host Gene Expression Assay for Early Detection of Respiratory Viral Infection: An Index-Cluster Prospective Cohort Study. Lancet Infect. Dis. 21 (3), 396–404. doi: 10.1016/S1473-3099(20)30486-2 32979932 PMC7515566

[B17] Muddassar H. Ke L. Muhammad N. A. Abdul W. Chenxi L. Di D.. (2020). A Viral Metagenomic Analysis Reveals Rich Viral Abundance and Diversity in Mosquitoes From Pig Farms. Transbound Emerg. Dis. 67 (1), 328–343. doi: 10.1111/tbed.13355 31512812

[B18] Pengpeng X. Chenghui L. Ying Z. Jicheng H. Xiaofang G. Lv X.. (2018). Metagenomic Sequencing From Mosquitoes in China Reveals a Variety of Insect and Human Viruses. Front. Cell. Infect. Microbiol. 8. doi: 10.3389/fcimb.2018.00364 PMC6202873 30406041

[B19] Priscilla Y. L. T. Leonela C. P. Sebald A. N. V. Jessica P. Andres M. Philip T. L.. (2020). Cas13b-Dependent and Cas13b-Independent RNA Knockdown of Viral Sequences in Mosquito Cells Following Guide RNA Expression. Commun. Biol. 3 (1), 413. doi: 10.1038/s42003-020-01142-6 32737398 PMC7395101

[B20] Ptasinska A. Whalley C. Bosworth A. Poxon C. Bryer C. Machin N.. (2021). Diagnostic Accuracy of Loop-Mediated Isothermal Amplification Coupled to Nanopore Sequencing (LamPORE) for the Detection of SARS-CoV-2 Infection at Scale in Symptomatic and Asymptomatic Populations. Clin. Microbiol. Infect. 27 (9), 1348.e1–1348.e7. doi: 10.1016/j.cmi.2021.04.008 33901668 PMC8064897

[B21] Rui-Chen L. Chang-Qiang Z. Chun-Hui W. Le-le A. Heng L. Bing Z.. (2020). Genetic Diversity and Population Structure of Aedes Aegypti After Massive Vector Control for Dengue Fever Prevention in Yunnan Border Areas. Sci. Rep. 10 (1), 12731. doi: 10.1038/s41598-020-69668-7 32728176 PMC7391764

[B22] Sarah L. Laura H. Kirsty E. Roger H. Anthony R. F. Daniel L. H.. (2021). Detection of Rift Valley Fever Virus RNA in Formalin-Fixed Mosquitoes by *In Situ* Hybridization (RNAscope®). Viruses 13 (6), 1079. doi: 10.3390/v13061079 34198809 PMC8227582

[B23] Yuan F. Xi-Shang L. Wei Z. Jing-Bo X. Jia-Zhi W. Shou-Qin Y.. (2021). Molecular Epidemiology of Mosquito-Borne Viruses at the China-Myanmar Border: Discovery of a Potential Epidemic Focus of Japanese Encephalitis. Infect. Dis. Poverty. 10 (1), 57. doi: 10.1186/s40249-021-00838-z 33902684 PMC8073957

